# Editorial: Food sustainability: challenges and opportunities for the future

**DOI:** 10.3389/fnut.2023.1341427

**Published:** 2024-01-05

**Authors:** Monika Thakur, Vinod K. Modi, Simin Feng

**Affiliations:** ^1^Amity Institute of Food Technology, Amity University Uttar Pradesh, Noida, India; ^2^College of Food Science and Engineering, Zhejiang University of Technology, Hangzhou, China

**Keywords:** food processing, food safety, food waste, valorization, food security, sustainable diets, food function, food culture

Food System (FS) encompasses a set of inter-connected processes and activities that include the cultivation, processing, distribution, consumption, disposal, and value-addition of food products made from various sources as: microorganisms, plants, and animals. The FS is composed of different sub-systems (e.g., farming system, waste management system, input supply system, etc.) and have interaction with other key systems as energy system, trade system, health system, etc. Sustainable Food Systems (SFS) provides nutrition to the masses while influencing their social, economic, and environmental foundations in order to produce nourishing food security for future generations. Its primary components are mainly Food safety and security. Thus, SFS creates a strong robust system with wide societal advantages while focusing upon economic, social, and environmental sustainability. The right of everyone to have access to wholesome food has been reaffirmed by world leaders. With an increasing worldwide population comes a greater need for innovation and increased efforts to process and raise agricultural production in a sustainable manner, enhance the global food supply chain, reduce food loss and waste, and guarantee that everyone has access to a healthy diet.

The foundation of the Sustainable Development Goals (SDGs) of the UN is a sustainable food system. The SDGs were well adopted in 2015, and targets the major transformations in agriculture and food systems in order to end hunger, achieve food security and improve nutrition by 2030. Sustainable food processing technologies; food waste and byproduct valuation; food diversity and security; advances in food formulations; sustainable diets and responsible consumption; preservation and promotion of traditional food cultures are the main components of the SFS (1).

The SFS development takes a comprehensive approach to examining Food Sustainability. The fundamental tenet of food sustainability is using natural resources sustainably for a longer time period without posing a threat to the environment or public health. The three major aspects of Food Sustainability as:

- Economic impacts
- Social impacts
- Environmental impacts.

The study's focus is on Food Sustainability, including its potential benefits, challenges and opportunities for the future (Figure 1). The most recent findings on the theme have been further elaborated by the different researchers as:

**Figure 1:**
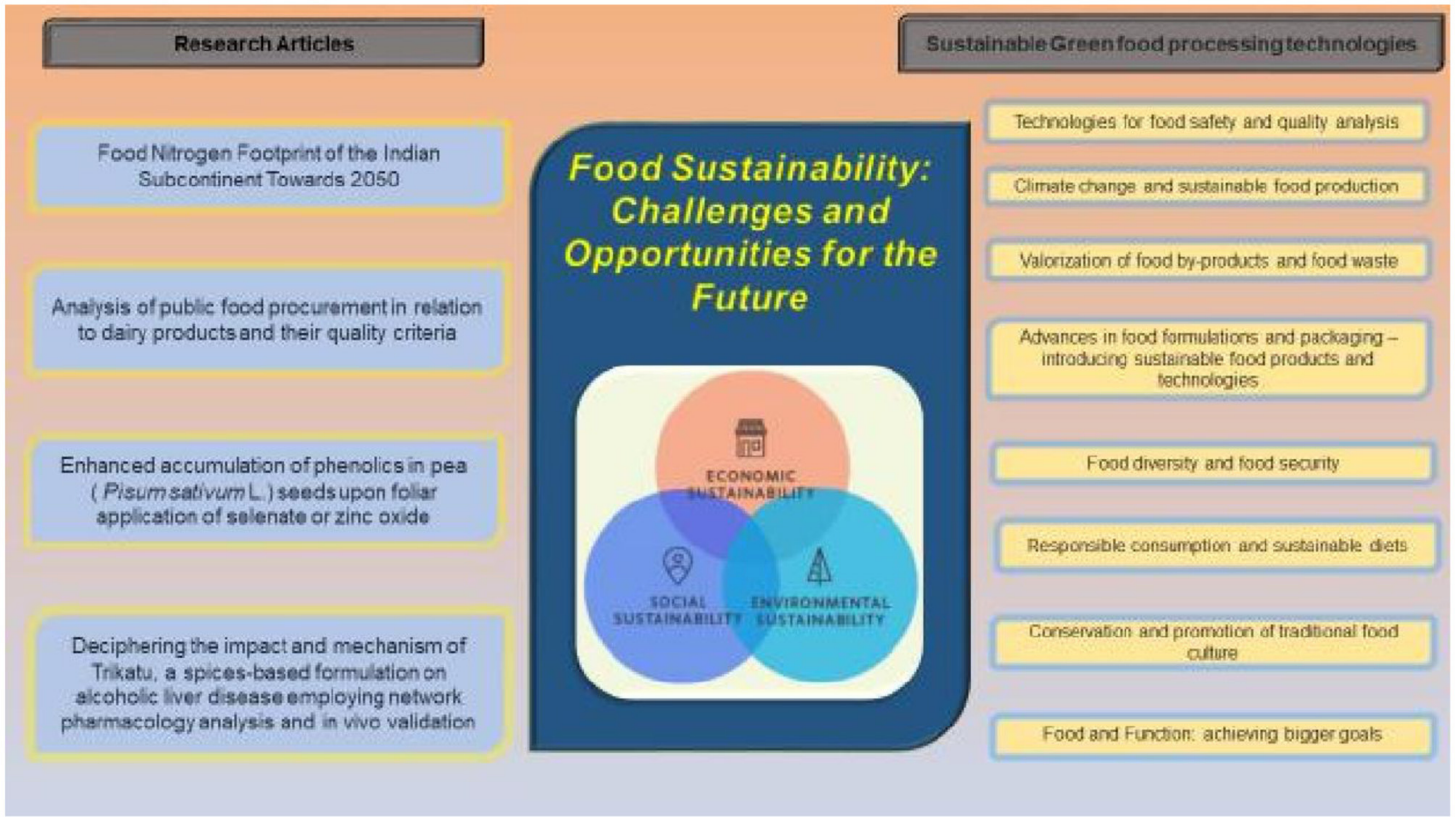
Food sustainability with different parameters for challenges and opportunities for the future.

Dhar et al. showed the Food Nitrogen Footprint of the Indian Subcontinent toward 2050. The main factors influencing the footprints are the Nitrogen Use Efficiency (NUE) of crop cultivation and religious dietary cultures. Buddhists had the lowest footprint over the period. An increase in the NUE of the crop cultivation and an altered diet results in a 13% reduction in the N footprint compared to the business-as-usual scenario. Dhar et al. conclude that improved crop cultivation NUEs and an altered religion-specific healthy diet would help in reducing the N footprints.

Sharma et al. deciphering the impact and mechanism of *Trikatu*, a spices-based formulation on Alcoholic Liver Disease (ALD) employing network pharmacology analysis and *in vivo* validation. *Trikatu Churna* (TC) comprising *Zingiber officinale* rhizome, *Piper longum*, and *Piper nigrum* fruit, is effective in treating liver diseases and has high nutraceutical values. This study evaluated the hepato-protective effects of different doses of TC as well as to identify the bioactive components and determine their mechanism of action against ethanol-induced ALD.

Malka et al. studied the enhanced accumulation of phenolic compounds in pea (*Pisum sativum* L.) seeds upon foliar application of selenate or zinc oxide. This study highlights the potential of foliar biofortification with trace elements for producing pea/pea products rich in bioactive plant metabolites beneficial for human health.

Brukało et al. documented the analysis of public food procurement in relation to dairy products and their quality criteria. Introducing minimum standards for the descriptions of dairy products in terms of organoleptic characteristics, composition features, and sustainability criteria will improve the quality of dairy products supplied to public institutions, particularly schools and kindergartens.

The Research Topic entitled “*Food sustainability: challenges and opportunities for the future*” presents a comprehensive collection of original research, reviews, and market reviews that encompass various facets of recent advancements in the field. The readers will recognize the diverse nature of the challenges and opportunities to explore the new avenues of research, thereby contributing to the development of SFS. Such efforts align with the SDG's play a pivotal and crucial role in enhancing production, nutrition, environmental sustainability, and overall quality of life.

The accuracy of the facts and concepts have been confirmed by the contributing authors. FS invariably require a more sustainable, comprehensive, and coordinated approach that places a strong emphasis on addressing the challenges they face and capitalizing on the opportunities they offer. A Sustainable Food System is central to the achievement of the SDGs, which serve as a guiding framework for present and future generations in pursuit of global sustainability. The SDGs are a set of 17 global goals established by the United Nations to address various social, economic, and environmental challenges by 2030.

Among the 17 SDGs, SDG-2, “*Zero Hunger”* is dedicated to attaining food security, eradicating all forms of malnutrition, and fostering sustainable agriculture. Additionally, SDG-12 underscores the importance of responsible production and consumption. To successfully realize these objectives, we must engage in introspection regarding current and future food production, meeting global nutritional and health needs. Consequently, it is imperative to integrate science and technology to promote food sustainability, including strategies that bolster food security, enhance nutrition, and advocate for sustainable agricultural practices.

A more comprehensive and coordinated strategy is necessary due to the complexity of food systems. Many issues related to food security and nutrition are complicated issues that cross institutional, divisional, and disciplinary lines and have contentious solutions. These difficulties arise in the context of increasingly interconnected food systems at many dimensions and levels. They necessitate integrated actions from all the stakeholders operating at the local, regional, national, and international levels on a variety of fronts, including trade, policy, health, the environment, gender norms, education, transportation, and infrastructure, in addition to agriculture. Instead of the ideas coming from these many angles colliding destructively, there has to be a synergistic blending (2).

Therefore, understanding the challenges and opportunities in food science and technology is vital because it directly impacts our ability to achieve the SDGs, particularly those related to food security, health, and sustainability. By harnessing innovation and research in this field, we can work toward creating more efficient, sustainable, and equitable food systems, thereby advancing the broader global goals of the SDGs by 2030.

## Author contributions

MT: Writing – original draft, Writing – review & editing. VM: Writing – review & editing. SF: Writing – original draft, Writing – review & editing.
